# Crystal structure of 1-benzyl-4-(2,4-di­chloro­phenyl)-2-imino-1,2,5,6,7,8,9,10-octa­hydro­cyclo­octa­[*b*]pyridine-3-carbo­nitrile

**DOI:** 10.1107/S1600536814023071

**Published:** 2014-10-31

**Authors:** R. A. Nagalakshmi, J. Suresh, S. Maharani, R. Ranjith Kumar, P. L. Nilantha Lakshman

**Affiliations:** a Department of Physics, The Madura College, Madurai 625 011, India; b Department of Organic Chemistry, School of Chemistry, Madurai Kamaraj University, Madurai 625 021, India; c Department of Food Science and Technology, University of Ruhuna, Mapalana, Kamburupitiya 81100, Sri Lanka

**Keywords:** crystal structure, cyclo­octa pyridine, imine

## Abstract

In the title compound, C_25_H_23_Cl_2_N_3_, the cyclo­octene ring adopts a twist chair–chair conformation. The dihedral angles between the central pyridine ring (r.m.s. deviation = 0.013 Å) and the pendant chloro­benzene and benzyl rings are 78.07 (11) and 87.47 (12)°, respectively. No directional inter­actions could be identified in the crystal and the packing is governed by van der Waals inter­actions.

## Abstract 2

In the title compound, the cyclo­octene ring adopts a twist chair–chair conformation. No directional inter­actions could be identified in the crystal and the packing is governed by van der Waals inter­actions.

## Chemical context   

Synthetic and naturally occurring pyridine derivatives have a broad range of biological activities (Thorat *et al.*, 2013 ▶), including anti­cancer and anti­microbial (Abdel-Megeed *et al.*, 2012 ▶) and anti­coagulant (de Candia *et al.*, 2013 ▶) properties. They also have numerous applications in medicinal chemistry (Passannanti *et al.*, 1998 ▶). The naturally occurring B6-vitamins pyridoxine, pyrodoxal, pyridoxamine and codeca­rbaxylase contain a pyridine nucleus (Shankaraiah *et al.*, 2010 ▶). The study of the properties and the formation of imines is of great interest due to the role they play in several important chemical and biological processes (Larkin, 1990 ▶). Imines and their complexes have a variety of applications in biological, clinical and analytical fields (Singh *et al.*, 1975 ▶; Patel *et al.*, 1999 ▶). Many pyridine-2-one and 3-cyano-2-imino pyridine derivatives exhibit anti­proliferative activity (McNamara & Cook, 1987 ▶; Abadi *et al.*,1998 ▶). As part of our ongoing studies of substituted pyridine systems (Vishnupriya *et al.*, 2014*a* ▶,*b* ▶), we now describe herein the synthesis and crystal structure of the title compound, (I).
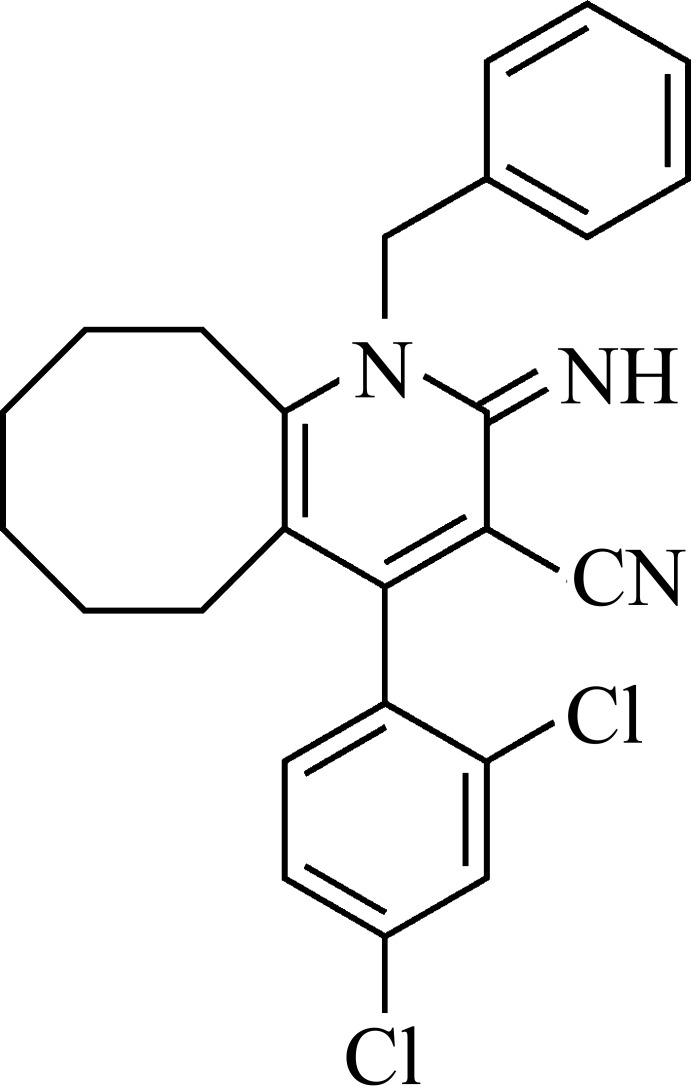


## Structural commentary   

**Figure 1:**
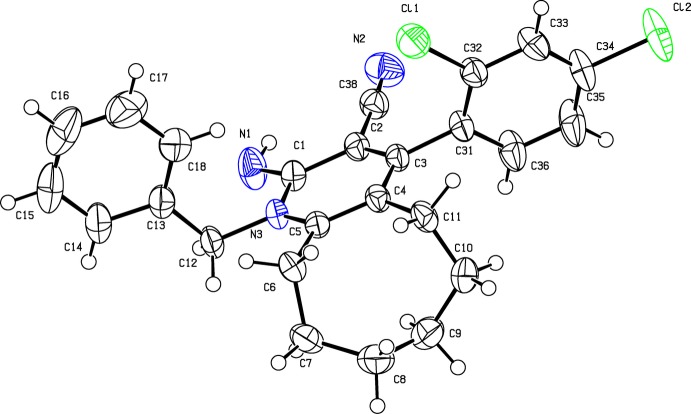
The mol­ecular structure of (I), showing 50% probability displacement ellipsoids and the atom-numbering scheme.

The mol­ecular structure of (I) is shown in Fig. 1 ▶. The cyclo­octane ring adopts a twisted chair–chair conformation. Steric hindrance rotates the phenyl (C13–C18) and aromatic (C31–C36) rings out of the plane of the central pyridine ring by 87.47 (12) and 78.07 (11)°, respectively. The imino group is nearly coplanar with the pyridine ring as indicated by the torsion angle N1—C1—N3—C5 = −179.8 (2)°. The C—C and C—N bond lengths [C1—C2 = 1.453 (3), C4—C3 = 1.416 (3), C5—N3 = 1.376 (2) and C1—N3 = 1.398 (3) Å] are shorter than the standard C—C and C—N bond lengths (1.54 and 1.47 Å, respectively), while the C=C bond lengths [C4=C5 = 1.374 (3) and C2=C3 = 1.367 (3) Å] are longer than the standard C=C bond (1.34 Å). This shows that there is a homo-conjugation effect on the pyridine ring. The C38—C2 (C*sp* ^2^—C*sp*) single bond [1.432 (3) Å] tends towards an aromatic bond length rather than a σ bond length (1.50 Å), presumably due to conjugation.

## Supra­molecular features   

No short directional contacts are observed in the crystal structure of (I) and the packing is governed by van der Waals inter­actions.

## Database survey   

Similar structures reported in the literature are 2-meth­oxy-4-(2-meth­oxy­phen­yl)-5,6,7,8,9,10-hexa­hydro­cyclo­octa­[*b*]pyridine-3-carbo­nitrile (Vishnupriya *et al.*, 2014*a* ▶), 4-(2-fluoro­phen­yl)-2-meth­oxy-5,6,7,8,9,10-hexa­hydro­cycloοcta[*b*]pyridine-3-carbo­nitrile (Vishnupriya *et al.*, 2014*b* ▶) and 1-benzyl-4-(4-chloro­phen­yl)-2-imino-1,2,5,6,7,8,9,10-octa­hydro­cyclo­octa[*b*]pyridine-3-carbo­nitrile (Nagalakshmi *et al.*, 2014 ▶). In the structure of (I) reported here, the *d-*planar conformation of the pyridine ring is similar to those found in related structures (Vishnupriya *et al.*, 2014*a* ▶,*b* ▶). There are no significant intra­molecular inter­actions or inter­molecular C—H⋯N inter­actions, as in the case of the related structures (Vishnupriya *et al.*, 2014*a* ▶,*b* ▶). In the title compound, the bond lengths in the central pyridine ring span the range 1.367 (3)–1.453 (3) Å, which compares well with the range (1.369–1.447 Å) observed in a similar structure (Nagalakshmi *et al.*, 2014 ▶), but these bonds are systematically longer in the title compound, due to the substitution by Cl atoms in the aromatic ring.

## Synthesis and crystallization   

A mixture of cyclo­octa­none (1 mmol), 2,4 dicholoro­benzaldehyde (1 mmol) and malono­nitrile (1 mmol) was taken in ethanol (10 ml) to which pTSA (*p*-toluene­sulfonic acid) (0.5 mmol) was added. The reaction mixture was heated under reflux for 2–3 h. After completion of the reaction (TLC), the reaction mixture was poured into crushed ice and extracted with ethyl acetate. The excess solvent was removed under vacuum and the residue was subjected to column chromatography using a petroleum ether/ethyl acetate mixture (97:3 *v*/*v*) as eluent to afford pure product. The product was recrystallized from ethyl acetate solution, affording colourless blocks. Melting point: 407 K, yield: 65%.

## Refinement   

C-bound H atoms were placed in calculated positions and allowed to ride on their carrier atoms, with C—H = 0.93 (aromatic CH) or 0.97 Å (methyl­ene CH_2_). Imine atom H1 was found in a difference map and refined freely, with the N—H distance restrained to 0.84 (2) Å. Isotropic displacement parameters for H atoms were calculated as *U*_iso_(H) = 1.2*U*_eq_(C) for CH and CH_2_ groups, while the *U*_iso_ factor for H1 was refined. Crystal data, data collection and structure refinement details are summarized in Table 1 ▶.

## Supplementary Material

Crystal structure: contains datablock(s) global, I. DOI: 10.1107/S1600536814023071/hb7305sup1.cif

Structure factors: contains datablock(s) I. DOI: 10.1107/S1600536814023071/hb7305Isup2.hkl

Supporting information file. DOI: 10.1107/S1600536814023071/hb7305Isup3.cml

CCDC reference: 1030164

Additional supporting information:  crystallographic information; 3D view; checkCIF report

## Figures and Tables

**Figure 2:**
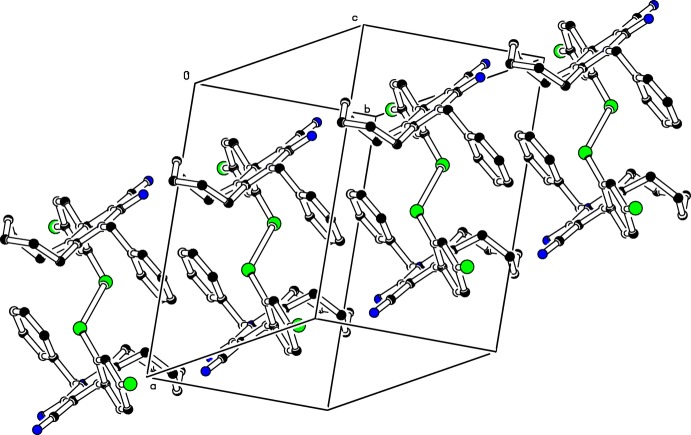
Partial packing diagram of the title compound. For clarity, H atoms are not shown.

**Table 1:** Experimental details

Crystal data	
Chemical formula	C_25_H_23_Cl_2_N_3_
*M*_r_	436.36
Crystal system, space group	Monoclinic, *P*2_1_/*n*
Temperature (K)	293
*a*, *b*, *c* ()	13.0297(6), 8.5901(3), 19.7449(8)
()	98.337(1)
*V* (^3^)	2186.62(15)
*Z*	4
Radiation type	Mo *K*
(mm^1^)	0.31
Crystal size (mm)	0.21 0.19 0.18

Data collection	
Diffractometer	Bruker Kappa APEXII
Absorption correction	Multi-scan (*SADABS*; Bruker, 2004 ▶)
*T*_min_, *T*_max_	0.967, 0.974
No. of measured, independent and observed [*I* > 2(*I*)] reflections	24005, 4762, 3607
*R*_int_	0.021
(sin /)_max_ (^1^)	0.639

Refinement	
*R*[*F* ^2^ > 2(*F* ^2^)], *wR*(*F* ^2^), *S*	0.051, 0.151, 1.05
No. of reflections	4762
No. of parameters	275
No. of restraints	1
H-atom treatment	H atoms treated by a mixture of independent and constrained refinement
_max_, _min_ (e ^3^)	0.65, 0.55

Computer programs: *APEX2* and *SAINT* (Bruker, 2004 ▶), *SHELXS97* and *SHELXL2014*/6 (Sheldrick, 2008 ▶) and *PLATON* (Spek, 2009 ▶).

## supplementary crystallographic information

### Crystal data

**Table:**

C_25_H_23_Cl_2_N_3_	*F*(000) = 912
*M**_r_* = 436.36	*D*_x_ = 1.326 Mg m^−^^3^
Monoclinic, *P*2_1_/*n*	Mo *K*α radiation, λ = 0.71073 Å
*a* = 13.0297 (6) Å	Cell parameters from 2000 reflections
*b* = 8.5901 (3) Å	θ = 2–31°
*c* = 19.7449 (8) Å	µ = 0.31 mm^−^^1^
β = 98.337 (1)°	*T* = 293 K
*V* = 2186.62 (15) Å^3^	Block, colourless
*Z* = 4	0.21 × 0.19 × 0.18 mm

### Data collection

**Table:**

Bruker Kappa APEXII diffractometer	3607 reflections with *I* > 2σ(*I*)
Radiation source: fine-focus sealed tube	*R*_int_ = 0.021
ω and φ scans	θ_max_ = 27.0°, θ_min_ = 2.1°
Absorption correction: multi-scan (*SADABS*; Bruker, 2004)	*h* = −16→16
*T*_min_ = 0.967, *T*_max_ = 0.974	*k* = −10→10
24005 measured reflections	*l* = −25→25
4762 independent reflections	

### Refinement

**Table:**

Refinement on *F*^2^	1 restraint
Least-squares matrix: full	Hydrogen site location: mixed
*R*[*F*^2^ > 2σ(*F*^2^)] = 0.051	H atoms treated by a mixture of independent and constrained refinement
*wR*(*F*^2^) = 0.151	*w* = 1/[σ^2^(*F*_o_^2^) + (0.0654*P*)^2^ + 1.5156*P*] where *P* = (*F*_o_^2^ + 2*F*_c_^2^)/3
*S* = 1.05	(Δ/σ)_max_ < 0.001
4762 reflections	Δρ_max_ = 0.65 e Å^−^^3^
275 parameters	Δρ_min_ = −0.55 e Å^−^^3^

### Special details

**Table:**

Geometry. All e.s.d.'s (except the e.s.d. in the dihedral angle between two l.s. planes) are estimated using the full covariance matrix. The cell e.s.d.'s are taken into account individually in the estimation of e.s.d.'s in distances, angles and torsion angles; correlations between e.s.d.'s in cell parameters are only used when they are defined by crystal symmetry. An approximate (isotropic) treatment of cell e.s.d.'s is used for estimating e.s.d.'s involving l.s. planes.

### Fractional atomic coordinates and isotropic or equivalent isotropic displacement parameters (Å^2^)

**Table:**

	*x*	*y*	*z*	*U*_iso_*/*U*_eq_	
C1	0.20063 (16)	0.4628 (2)	0.12039 (10)	0.0359 (4)	
C2	0.18748 (15)	0.4805 (2)	0.04645 (10)	0.0330 (4)	
C3	0.24407 (15)	0.3969 (2)	0.00591 (9)	0.0312 (4)	
C4	0.31948 (15)	0.2887 (2)	0.03557 (9)	0.0321 (4)	
C5	0.33572 (15)	0.2735 (2)	0.10560 (9)	0.0307 (4)	
C6	0.41621 (16)	0.1647 (3)	0.14117 (10)	0.0378 (5)	
H6A	0.4428	0.2077	0.1857	0.045*	
H6B	0.4735	0.1591	0.1149	0.045*	
C7	0.3769 (2)	−0.0005 (3)	0.15108 (12)	0.0494 (6)	
H7A	0.4257	−0.0518	0.1857	0.059*	
H7B	0.3113	0.0069	0.1687	0.059*	
C8	0.3611 (2)	−0.1035 (3)	0.08746 (14)	0.0609 (7)	
H8A	0.4264	−0.1091	0.0694	0.073*	
H8B	0.3449	−0.2078	0.1015	0.073*	
C9	0.2775 (2)	−0.0542 (3)	0.02966 (14)	0.0605 (7)	
H9A	0.2413	−0.1468	0.0110	0.073*	
H9B	0.2276	0.0100	0.0489	0.073*	
C10	0.3141 (2)	0.0348 (3)	−0.02886 (12)	0.0520 (6)	
H10A	0.2540	0.0604	−0.0620	0.062*	
H10B	0.3579	−0.0335	−0.0514	0.062*	
C11	0.37366 (17)	0.1839 (3)	−0.00902 (10)	0.0399 (5)	
H11A	0.4417	0.1575	0.0151	0.048*	
H11B	0.3835	0.2398	−0.0503	0.048*	
C12	0.29562 (17)	0.3405 (3)	0.22118 (9)	0.0398 (5)	
H12A	0.3144	0.2335	0.2327	0.048*	
H12B	0.2314	0.3628	0.2387	0.048*	
C13	0.37932 (18)	0.4462 (3)	0.25617 (11)	0.0414 (5)	
C14	0.3998 (2)	0.4435 (3)	0.32736 (12)	0.0560 (7)	
H14	0.3629	0.3757	0.3516	0.067*	
C15	0.4736 (3)	0.5392 (4)	0.36243 (16)	0.0791 (10)	
H15	0.4858	0.5369	0.4100	0.095*	
C16	0.5294 (3)	0.6381 (4)	0.3273 (2)	0.0870 (11)	
H16	0.5793	0.7030	0.3510	0.104*	
C17	0.5117 (3)	0.6416 (4)	0.25697 (19)	0.0786 (9)	
H17	0.5498	0.7083	0.2331	0.094*	
C18	0.4366 (2)	0.5451 (3)	0.22166 (14)	0.0576 (7)	
H18	0.4249	0.5475	0.1741	0.069*	
C31	0.22607 (16)	0.4217 (3)	−0.06963 (9)	0.0358 (4)	
C32	0.29212 (17)	0.5111 (3)	−0.10249 (11)	0.0397 (5)	
C33	0.27671 (19)	0.5318 (3)	−0.17275 (11)	0.0485 (6)	
H33	0.3221	0.5922	−0.1939	0.058*	
C34	0.19288 (19)	0.4609 (4)	−0.21027 (11)	0.0534 (6)	
C35	0.1247 (2)	0.3726 (4)	−0.18008 (12)	0.0650 (8)	
H35	0.0677	0.3265	−0.2064	0.078*	
C36	0.14182 (18)	0.3531 (4)	−0.10991 (11)	0.0547 (7)	
H36	0.0960	0.2927	−0.0892	0.066*	
C38	0.11331 (17)	0.5948 (3)	0.01833 (11)	0.0395 (5)	
N1	0.15090 (18)	0.5350 (3)	0.16254 (10)	0.0566 (6)	
N2	0.05483 (17)	0.6890 (3)	−0.00069 (12)	0.0586 (6)	
N3	0.27777 (13)	0.3564 (2)	0.14615 (8)	0.0333 (4)	
Cl1	0.39949 (6)	0.59550 (9)	−0.05516 (3)	0.0674 (2)	
Cl2	0.17481 (6)	0.48449 (13)	−0.29858 (3)	0.0841 (3)	
H1	0.109 (2)	0.598 (3)	0.1392 (14)	0.097 (12)*	

### Atomic displacement parameters (Å^2^)

**Table:**

	*U*^11^	*U*^22^	*U*^33^	*U*^12^	*U*^13^	*U*^23^
C1	0.0379 (11)	0.0406 (11)	0.0286 (9)	0.0020 (9)	0.0026 (8)	−0.0034 (8)
C2	0.0313 (10)	0.0384 (11)	0.0284 (9)	−0.0006 (8)	0.0010 (7)	0.0008 (8)
C3	0.0303 (9)	0.0384 (11)	0.0241 (9)	−0.0070 (8)	0.0012 (7)	0.0010 (8)
C4	0.0318 (10)	0.0381 (11)	0.0270 (9)	−0.0027 (8)	0.0064 (7)	0.0003 (8)
C5	0.0307 (9)	0.0338 (10)	0.0275 (9)	−0.0021 (8)	0.0036 (7)	−0.0001 (8)
C6	0.0373 (11)	0.0450 (12)	0.0305 (10)	0.0037 (9)	0.0026 (8)	0.0048 (9)
C7	0.0635 (15)	0.0448 (13)	0.0420 (12)	0.0054 (11)	0.0147 (11)	0.0117 (10)
C8	0.089 (2)	0.0399 (13)	0.0577 (15)	−0.0023 (13)	0.0241 (14)	0.0028 (12)
C9	0.0794 (19)	0.0506 (15)	0.0532 (15)	−0.0222 (14)	0.0155 (14)	−0.0092 (12)
C10	0.0692 (16)	0.0524 (14)	0.0359 (11)	−0.0017 (12)	0.0128 (11)	−0.0112 (10)
C11	0.0470 (12)	0.0480 (12)	0.0274 (9)	0.0036 (10)	0.0144 (8)	0.0022 (9)
C12	0.0493 (12)	0.0488 (13)	0.0213 (9)	0.0015 (10)	0.0049 (8)	−0.0001 (8)
C13	0.0469 (12)	0.0432 (12)	0.0324 (10)	0.0105 (10)	−0.0001 (9)	−0.0052 (9)
C14	0.0644 (16)	0.0678 (17)	0.0335 (11)	0.0078 (13)	−0.0010 (11)	−0.0101 (11)
C15	0.084 (2)	0.102 (3)	0.0453 (15)	−0.001 (2)	−0.0122 (15)	−0.0267 (16)
C16	0.081 (2)	0.086 (2)	0.085 (2)	−0.0124 (19)	−0.0171 (19)	−0.037 (2)
C17	0.079 (2)	0.0652 (19)	0.089 (2)	−0.0210 (17)	0.0023 (18)	−0.0037 (17)
C18	0.0681 (17)	0.0525 (15)	0.0493 (14)	−0.0066 (13)	−0.0015 (12)	0.0008 (12)
C31	0.0341 (10)	0.0476 (12)	0.0249 (9)	−0.0008 (9)	0.0018 (8)	0.0027 (8)
C32	0.0392 (11)	0.0461 (12)	0.0322 (10)	−0.0047 (9)	0.0001 (8)	0.0042 (9)
C33	0.0483 (13)	0.0616 (15)	0.0370 (11)	−0.0005 (11)	0.0106 (10)	0.0126 (11)
C34	0.0502 (14)	0.0861 (19)	0.0228 (10)	0.0064 (13)	0.0016 (9)	0.0038 (11)
C35	0.0443 (13)	0.116 (3)	0.0318 (12)	−0.0175 (15)	−0.0042 (10)	−0.0060 (14)
C36	0.0392 (12)	0.092 (2)	0.0319 (11)	−0.0169 (13)	0.0006 (9)	0.0004 (12)
C38	0.0366 (11)	0.0438 (12)	0.0369 (11)	−0.0021 (10)	0.0010 (9)	0.0026 (9)
N1	0.0649 (14)	0.0694 (15)	0.0354 (10)	0.0265 (12)	0.0070 (9)	−0.0081 (10)
N2	0.0502 (12)	0.0571 (13)	0.0659 (14)	0.0074 (11)	−0.0001 (10)	0.0140 (11)
N3	0.0371 (9)	0.0409 (9)	0.0213 (7)	0.0015 (7)	0.0023 (6)	−0.0011 (7)
Cl1	0.0692 (4)	0.0768 (5)	0.0511 (4)	−0.0388 (4)	−0.0089 (3)	0.0102 (3)
Cl2	0.0784 (5)	0.1477 (8)	0.0242 (3)	0.0001 (5)	0.0007 (3)	0.0090 (4)

### Geometric parameters (Å, º)

**Table:**

C1—N1	1.286 (3)	C12—N3	1.472 (2)
C1—N3	1.398 (3)	C12—C13	1.507 (3)
C1—C2	1.453 (3)	C12—H12A	0.9700
C2—C3	1.367 (3)	C12—H12B	0.9700
C2—C38	1.432 (3)	C13—C18	1.375 (4)
C3—C4	1.416 (3)	C13—C14	1.392 (3)
C3—C31	1.491 (2)	C14—C15	1.374 (4)
C4—C5	1.374 (3)	C14—H14	0.9300
C4—C11	1.505 (3)	C15—C16	1.371 (5)
C5—N3	1.376 (2)	C15—H15	0.9300
C5—C6	1.501 (3)	C16—C17	1.374 (5)
C6—C7	1.531 (3)	C16—H16	0.9300
C6—H6A	0.9700	C17—C18	1.390 (4)
C6—H6B	0.9700	C17—H17	0.9300
C7—C8	1.526 (4)	C18—H18	0.9300
C7—H7A	0.9700	C31—C32	1.383 (3)
C7—H7B	0.9700	C31—C36	1.390 (3)
C8—C9	1.519 (4)	C32—C33	1.384 (3)
C8—H8A	0.9700	C32—Cl1	1.725 (2)
C8—H8B	0.9700	C33—C34	1.370 (4)
C9—C10	1.519 (3)	C33—H33	0.9300
C9—H9A	0.9700	C34—C35	1.369 (4)
C9—H9B	0.9700	C34—Cl2	1.737 (2)
C10—C11	1.519 (3)	C35—C36	1.381 (3)
C10—H10A	0.9700	C35—H35	0.9300
C10—H10B	0.9700	C36—H36	0.9300
C11—H11A	0.9700	C38—N2	1.138 (3)
C11—H11B	0.9700	N1—H1	0.8599 (10)
			
N1—C1—N3	118.88 (19)	C10—C11—H11B	109.0
N1—C1—C2	127.1 (2)	H11A—C11—H11B	107.8
N3—C1—C2	114.05 (17)	N3—C12—C13	113.81 (18)
C3—C2—C38	121.57 (18)	N3—C12—H12A	108.8
C3—C2—C1	122.56 (18)	C13—C12—H12A	108.8
C38—C2—C1	115.84 (18)	N3—C12—H12B	108.8
C2—C3—C4	120.20 (17)	C13—C12—H12B	108.8
C2—C3—C31	119.39 (18)	H12A—C12—H12B	107.7
C4—C3—C31	120.41 (17)	C18—C13—C14	118.2 (2)
C5—C4—C3	118.32 (17)	C18—C13—C12	123.6 (2)
C5—C4—C11	121.01 (18)	C14—C13—C12	118.2 (2)
C3—C4—C11	120.47 (17)	C15—C14—C13	121.1 (3)
C4—C5—N3	121.26 (18)	C15—C14—H14	119.5
C4—C5—C6	121.66 (17)	C13—C14—H14	119.5
N3—C5—C6	117.07 (16)	C16—C15—C14	120.0 (3)
C5—C6—C7	114.38 (18)	C16—C15—H15	120.0
C5—C6—H6A	108.7	C14—C15—H15	120.0
C7—C6—H6A	108.7	C15—C16—C17	120.1 (3)
C5—C6—H6B	108.7	C15—C16—H16	120.0
C7—C6—H6B	108.7	C17—C16—H16	120.0
H6A—C6—H6B	107.6	C16—C17—C18	119.8 (3)
C8—C7—C6	116.11 (19)	C16—C17—H17	120.1
C8—C7—H7A	108.3	C18—C17—H17	120.1
C6—C7—H7A	108.3	C13—C18—C17	120.9 (3)
C8—C7—H7B	108.3	C13—C18—H18	119.6
C6—C7—H7B	108.3	C17—C18—H18	119.6
H7A—C7—H7B	107.4	C32—C31—C36	117.42 (19)
C9—C8—C7	116.9 (2)	C32—C31—C3	122.01 (18)
C9—C8—H8A	108.1	C36—C31—C3	120.56 (18)
C7—C8—H8A	108.1	C31—C32—C33	122.1 (2)
C9—C8—H8B	108.1	C31—C32—Cl1	119.34 (16)
C7—C8—H8B	108.1	C33—C32—Cl1	118.51 (17)
H8A—C8—H8B	107.3	C34—C33—C32	118.3 (2)
C10—C9—C8	116.2 (2)	C34—C33—H33	120.9
C10—C9—H9A	108.2	C32—C33—H33	120.9
C8—C9—H9A	108.2	C35—C34—C33	121.8 (2)
C10—C9—H9B	108.2	C35—C34—Cl2	119.97 (19)
C8—C9—H9B	108.2	C33—C34—Cl2	118.19 (19)
H9A—C9—H9B	107.4	C34—C35—C36	118.9 (2)
C9—C10—C11	115.67 (19)	C34—C35—H35	120.6
C9—C10—H10A	108.4	C36—C35—H35	120.6
C11—C10—H10A	108.4	C35—C36—C31	121.5 (2)
C9—C10—H10B	108.4	C35—C36—H36	119.3
C11—C10—H10B	108.4	C31—C36—H36	119.3
H10A—C10—H10B	107.4	N2—C38—C2	176.4 (2)
C4—C11—C10	112.94 (18)	C1—N1—H1	108 (2)
C4—C11—H11A	109.0	C5—N3—C1	123.54 (16)
C10—C11—H11A	109.0	C5—N3—C12	121.18 (17)
C4—C11—H11B	109.0	C1—N3—C12	115.27 (16)
			
N1—C1—C2—C3	−179.6 (2)	C15—C16—C17—C18	0.4 (6)
N3—C1—C2—C3	1.6 (3)	C14—C13—C18—C17	−1.1 (4)
N1—C1—C2—C38	2.2 (3)	C12—C13—C18—C17	179.0 (3)
N3—C1—C2—C38	−176.61 (18)	C16—C17—C18—C13	0.2 (5)
C38—C2—C3—C4	177.73 (19)	C2—C3—C31—C32	101.6 (2)
C1—C2—C3—C4	−0.4 (3)	C4—C3—C31—C32	−78.0 (3)
C38—C2—C3—C31	−1.9 (3)	C2—C3—C31—C36	−79.2 (3)
C1—C2—C3—C31	−179.99 (19)	C4—C3—C31—C36	101.2 (3)
C2—C3—C4—C5	−1.7 (3)	C36—C31—C32—C33	−0.5 (4)
C31—C3—C4—C5	177.90 (18)	C3—C31—C32—C33	178.7 (2)
C2—C3—C4—C11	173.25 (18)	C36—C31—C32—Cl1	−178.61 (19)
C31—C3—C4—C11	−7.1 (3)	C3—C31—C32—Cl1	0.6 (3)
C3—C4—C5—N3	2.5 (3)	C31—C32—C33—C34	0.1 (4)
C11—C4—C5—N3	−172.43 (18)	Cl1—C32—C33—C34	178.2 (2)
C3—C4—C5—C6	−178.29 (18)	C32—C33—C34—C35	0.6 (4)
C11—C4—C5—C6	6.8 (3)	C32—C33—C34—Cl2	−179.00 (19)
C4—C5—C6—C7	−89.6 (2)	C33—C34—C35—C36	−0.8 (5)
N3—C5—C6—C7	89.7 (2)	Cl2—C34—C35—C36	178.7 (2)
C5—C6—C7—C8	75.3 (3)	C34—C35—C36—C31	0.4 (5)
C6—C7—C8—C9	−65.1 (3)	C32—C31—C36—C35	0.3 (4)
C7—C8—C9—C10	98.2 (3)	C3—C31—C36—C35	−179.0 (3)
C8—C9—C10—C11	−57.6 (3)	C4—C5—N3—C1	−1.2 (3)
C5—C4—C11—C10	88.3 (2)	C6—C5—N3—C1	179.53 (18)
C3—C4—C11—C10	−86.5 (2)	C4—C5—N3—C12	−179.71 (19)
C9—C10—C11—C4	−49.5 (3)	C6—C5—N3—C12	1.1 (3)
N3—C12—C13—C18	−2.0 (3)	N1—C1—N3—C5	−179.8 (2)
N3—C12—C13—C14	178.0 (2)	C2—C1—N3—C5	−0.8 (3)
C18—C13—C14—C15	1.4 (4)	N1—C1—N3—C12	−1.2 (3)
C12—C13—C14—C15	−178.7 (3)	C2—C1—N3—C12	177.74 (17)
C13—C14—C15—C16	−0.8 (5)	C13—C12—N3—C5	86.2 (2)
C14—C15—C16—C17	−0.1 (6)	C13—C12—N3—C1	−92.4 (2)
